# Evaluating enterovirus diversity among symptomatic patients in Hungary during and after easing the COVID-19 lockdown

**DOI:** 10.1186/s12985-025-02835-2

**Published:** 2025-06-24

**Authors:** Nóra Deézsi-Magyar, Gyula Zsidei, Norbert Kiss, Bereniké Novák, Marianna Mezősi-Csaplár, Katalin Réka Tarcsai, Adrienne Lukács, Erzsébet Barcsay, Katalin Szomor, Mária Takács

**Affiliations:** 1 Department of Microbiological Reference Laboratories, National Center for Public Health and Pharmacy, Budapest, 1097 Hungary; 2 School of PhD Studies, Semmelweis University, Budapest, 1089 Hungary; 3 Institute of Medical Microbiology, Semmelweis University, Budapest, 1089 Hungary

**Keywords:** Enteroviruses, Genetic diversity, COVID-19 lockdown, Pandemic, Phylogenetic analysis

## Abstract

**Background:**

The COVID-19 pandemic led to widespread public health interventions that significantly affected the transmission of various pathogens, including enteroviruses (EVs). EVs exhibit considerable genetic diversity and can cause clinical manifestations ranging from mild illnesses to severe diseases. Our present study aimed to evaluate the diversity of circulating EV types in Hungary and assess the impact of lockdown measures on EV prevalence based on testing clinical samples obtained from symptomatic patients.

**Methods:**

As part of the routine enterovirus diagnosis, we conducted quantitative reverse transcription polymerase chain reaction (RT-qPCR) on clinical samples obtained from patients presenting with symptoms corresponding to EV infection. Positive samples were then subjected to virus isolation in cell culture and next-generation sequencing (NGS). Phylogenetic analysis was performed to place the newly generated sequences within the global diversity of EV strains for comparison.

**Results:**

During this period, an overall number of 125 patients tested positive for EVs, mostly children under the age of 15 years. The most common symptoms were fever, hand-foot-mouth disease, encephalitis, and meningitis. The temporal distribution of EV-positive cases showed strong seasonality, with peaks in the summer and autumn months. The lowest number of confirmed cases occurred during the lockdown years, attributed to limited sample collection and reduced personal contacts. However, following the easing of restrictions, the number of cases significantly increased, with the highest incidence observed in 2022. The distribution of EV genotypes shifted notably after easing the lockdowns. While only coxsackievirus (CV)A6 was detected during 2021, a broader range of genotypes emerged afterwards, including CVA10, CVA16, echovirus E9, and E11.

**Conclusions:**

Next-generation sequencing analysis revealed notable genotypic diversity, providing valuable insights into the evolution of EVs in Hungary and across Europe. These findings underscore the importance of continued surveillance of enterovirus infections, particularly in the context of pandemic recovery, as the shifting EV genotype landscape may impact disease severity and spread, highlighting the need for adaptive public health responses.

**Supplementary Information:**

The online version contains supplementary material available at 10.1186/s12985-025-02835-2.

## Introduction

Due to the rapidly increasing number of severe acute respiratory syndrome coronavirus 2 (SARS-CoV-2) infections worldwide, the World Health Organization (WHO) declared the Coronavirus Disease 2019 (COVID-19) outbreak as a pandemic on March 11, 2020 [1, 2]. In response to the extremely high number of confirmed cases and deaths, and burden on public health systems, strict interventions and countermeasures were implemented in most countries. These regulations included national lockdowns, restrictions of traveling, border closure and quarantine for returning travelers, school closures, widespread routine rapid testing, isolation of confirmed cases, and contact tracing. Individual control and prevention measures were also taken, such as social distancing and banning large gatherings, hand cleaning, and wearing masks. These collective regulations strongly influenced the spread of different SARS-CoV-2 variants and other air-borne diseases, and also pathogens that spread via droplets or even through the fecal-oral route, such as enteroviruses (EVs) [1, 2]. In Hungary, the restrictions and lockdown were introduced after the first identified SARS-CoV-2 cases in March 2020. Easing the lockdown was taking place until April 2021 introduced in multiple stages [3].

EVs (family *Picornaviridae*, genus *Enterovirus*) form a diverse family with over 300 geno- and/or serotypes, of which different clades co-circulate worldwide [4]. EV infections are frequently asymptomatic, but have the potential to cause mild to life threatening disease. EVs replicate primarily in the gastrointestinal tract and/or the upper respiratory tract, but they can also infect other tissues, e.g., nerve, muscle, etc. The wide range of clinical manifestations include flu-like symptoms, fever, meningitis, encephalitis, myelitis, myocarditis, herpangina and conjunctivitis. Some enterovirus species are associated with specific clinical syndromes such as respiratory infections (EVD68, Coxsackievirus B [CVB]), neurological infections (EVA71, echoviruses, Coxsackievirus A [CVA]), acute flaccid myelitis (AFM; EVD68) or acute flaccid paralysis (AFP; polioviruses) and hand-foot-mouth disease (HFMD; CVA and CVB) [5–8].

The EV genome is approximately 7,500 nucleotide in length, in the form of a single-stranded positive-sense RNA, encoding one large polyprotein in a single open reading frame (ORF), with a highly structured 5′-untranslated region (5′UTR) and 3′UTR with a poly(A) tail [9]. The encoded polyprotein is approximately 2,100 amino acids in length, and is further cleaved post-transcriptionally to form three precursor proteins (P1, P2, and P3). P1 (capsid region) precursor protein incorporates four structural viral proteins (VP1, VP2, VP3, and VP4). VP1, VP2, and VP3 are exposed on the capsid surface, while VP4 is present inside the virus capsid. P2 and P3 regions encode seven non-structural proteins (P2–2 A, 2B, 2 C and P3–3 A, 3B, 3 C, 3D), such as the RNA-dependent RNA polymerase, proteases, and other necessary proteins needed for intracellular virus replication [9, 10]. As VP1 (∼900 nucleotides) is the most external and immune-dominant capsid protein, the VP1 gene region serves for the basis of classification of EVs and phylogenetic analysis [10, 11].

Laboratory diagnosis of EVs are primarily based on direct virus detection by reverse-transcription polymerase chain reaction (RT-PCR) targeting the conserved 5’ UTR region. Virus isolation from clinical samples is also a sensitive method for direct detection. The most commonly used laboratory cell lines for EV isolation include human rhabdomyosarcoma (RD), human lung carcinoma (A549) and African green monkey kidney (Vero) cells. The most suitable clinical samples for EV diagnostics include stool, cerebrospinal fluid (CSF), nasopharyngeal aspirate/swab, vesicular fluid, bronchoalveolar lavage, blood, conjunctival swab, and biopsy specimen [9]. It is also important to mention, that reference laboratories for polioviruses (type EV-C) in accordance with the guidelines of the WHO operate a well-established system worldwide, in order to screen children under the age of 15 with symptoms of paralysis of non-traumatic origin (AFP surveillance), in order to rule out poliovirus as the pathogenic factor. AFP testing consists of direct nucleic acid detection and virus isolation in susceptible RD and L20B cells (transfected mouse L cells expressing the human CD155 receptor, selective cell line for polioviruses) from the stool samples of the symptomatic infant or child [12, 13].

During the last couple of years, next-generation sequencing (NGS) has been successfully applied in typing and identification. As certain EV genotypes have enhanced potential to recombine, frequent recombination events in enteroviruses can be identified and recombination breakpoints can be mapped by sequencing the whole viral genome. Furthermore, NGS enables genotyping of serologically untypable EVs and provides the capability to identify mutations and virulence markers across the whole genome [9].

As of May 2025, there are no licensed vaccines available in the European Union– apart from poliovirus vaccines– specifically targeting EVs. Currently, preventive measures against EV infections include supportive care. Public health strategies focus on hygiene education, early detection, and symptomatic treatment to manage outbreaks and reduce transmission. Considering, the aim of the present study was to evaluate the circulating enterovirus types among patients with a wide range of symptoms and assess the impact of the COVID-19 pandemic lockdown on the number of identified cases and on enterovirus genotype diversity in Hungary. Furthermore, our newly generated whole genome sequences and VP1 sequences can contribute to better understand the spatial and temporal evolution of enteroviruses across Europe and worldwide.

## Materials and methods

### Routine diagnosis of EV infection

The National Reference Laboratory for Enteroviruses at the National Center for Public Health and Pharmacy, Budapest carries out the routine enterovirus diagnosis in accordance with its obligation to provide in-area care in different regions of Hungary, and among hospitalized patients with severe neurological infections at the national level. Furthermore, the National WHO Poliovirus Reference Laboratory performs the AFP surveillance on the basis of WHO recommendations in accordance with the 18/1998. (VI. 3.) NM decree on the necessary epidemiological measures to prevent infectious diseases and epidemics [12, 13]. Between 2020 and 2024, stool, nasopharyngeal swab/wash, trachea, CSF, vesicular fluid, skin lesions, etc. samples were received from symptomatic cases from all over the country.

Fecal samples, prior to nucleic acid extraction were washed into Dulbecco modified Eagle medium (DMEM; VWR International bv, Leuven, Belgium, Cat# 392–0413) supplemented with 1% antibiotics. Viral RNA from clinical samples was extracted using the QIAamp Viral RNA Mini Kit (Qiagen GmbH, Hilden, Germany, Cat# 52904) according to the manufacturer’s instructions. Subsequent RT-qPCR was performed at the LightCycler 480 Instrument II platform (Hoffmann-La Roche, Basel, Switzerland) using the LightCycler Multiplex RNA Virus Master kit (Hoffmann-La Roche, Basel, Switzerland, Cat# 06 754 155 001). The applied primers and probes specific for the 5’ UTR genome region were previously described by Benschop et al. [14].

### Virus isolation in cell culture

Enterovirus-positive clinical samples with Ct ≤ 37 were subjected to virus isolation in RD cells (CDC, Atlanta, USA, acc. No. 081003). Cell monolayers at 50–70% confluence level seeded 24 h before infection in T25 flasks were used. The clear supernatant of the uninfected cell monolayers were aspired and the attached cells were washed in sterile phosphate-buffer saline (PBS). After, cells were inoculated with 500 µL of the clinical specimen. Fecal samples, prior to inoculation were pre-treated in 4 mL DMEM and 500 µL chloroform, mixed thoroughly and centrifuged at 5000 rpm for 20 min. The clear supernatant of the suspension was used for cell inoculation. After, flasks were incubated at 37 °C for 1 h, and then supplemented with 6 mL DMEM and 10% fetal bovine serum (FBS; Euroclone S.p.A., Pero, Italy, Cat# ECS0183L). Flasks were incubated for 5 days at 37 °C under 5% CO_2_. Positivity was assessed under the light microscope by visual evaluation of the cytopathic effect (CPE) compared to the controls. Negative samples were passaged further in RD cells.

### Sequencing and genotyping

Enterovirus-positive viral nucleic acid extracted directly from the clinical samples or from the supernatant of the virus isolates were subjected to sequence-independent single-primer amplification and subsequent next generation sequencing (SISPA-Seq) starting with DNase I digestion (Turbo DNAse; Invitrogen, Thermo Fisher Scientific, Waltham, Massachusetts, USA, Cat# AM2238) and AMPure XP bead (Beckman Coulter, Brea, California, USA, Cat# A63881) purification. Clear nucleic acid was eluted in 10 µL nuclease-free water. To reduce the abundance of host-derived ribosomal RNA, the RiboCop rRNA Depletion Kit HMR V2 (Lexogen GmbH, Vienna, Austria, Cat# 144) was used. Randomly amplified DNA products were prepared using the SISPA protocol with the RA01/RA01-N8 primers as previously described [15–17]. After enrichment, the Illumina Nextera XT V2 (Illumina, Waltham, Massachusetts, USA, Cat# 15032354) library preparation was performed. Sequencing was conducted on the Illumina MiSeq system (Illumina, Waltham, Massachusetts, USA, RRID: SCR_016379) instrument using 2 × 150 bp paired-end chemistry (Reagent Kit v2 Micro; Illumina, Waltham, Massachusetts, USA, Cat# MS-102-2002). Where the SISPA-Seq was unsuccessful, direct sequencing of the amplicons generated by conventional PCR targeting the 5’UTR was performed, following the protocol of Kapusinszky et al. [18].

### Data analysis

Descriptive data, including age and most commonly reported symptoms were assessed by using the GraphPad Prism 9.5.0 software (GraphPad Software Inc., Boston, MA, USA, RRID: SCR_002798). Visualization was carried out by using the GraphPad Prism 9.5.0 software.

For the analysis of the effect of COVID-19 lockdown and its easing on EV detection rates, the data was split into four time periods: May 2020 - April 2021 (COVID lockdown), May 2021 - April 2022, May 2022 - April 2023, and May 2023 - April 2024. Fisher’s exact test was used to compare the EV detection rates during the selected time periods with a significance level set at 5%. Calculations were performed in GraphPad Prism 9.5.0. To assess whether EV diversity significantly increased post-lockdown and to evaluate the age-dependency of genotype distributions, we employed the Shannon diversity index (SDI), representing a value that accounts for the number and relative frequency of genotypes [19]. Calculations were performed in Windows Excel.

To perform genome analysis, fastq files were processed in the Geneious Prime 2025.0.2 software (Biomatters, Auckland, New Zealand, RRID: SCR_010519). First, raw reads were quality trimmed by using BBduk plug-in (RRID: SCR_016969) and duplicate reads were removed. After, reads were mapped against a custom enterovirus database and the National Center for Biotechnology and Information (NCBI, RRID: SCR_004860) nucleotide database using the Kraken 2 tool (RRID: SCR_026838). Reads were then mapped against the best reference to assemble whole genome sequences or VP1 sequences. Consensus sequences were called with a threshold of bases corresponding at least 40% of the read sequences. To assign genotypes to the detected EV strains, the NCBI Blastn (RRID: SCR_001598) and RIVM Enterovirus Genotyping Tool was used [20].

Nucleotide alignment was performed using the Clustal Omega 1.2.2 multiple alignment tool (RRID: SCR_001591) installed within the Geneious Prime 2025.0.2 software [21]. Sequences from the Genbank were downloaded to perform phylogenetic analysis based on the VP1 region of the identified EV genotypes (CVA6, EVA71, CVB5, CVB4, and E6). The Maximum Likelihood method of MEGA X v10.0.5 software (Pennsylvania State University, USA, RRID: SCR_000667) was used to determine the best-fit model of nt substitution [22]. Phylogenetic tree construction based on the VP1 region was performed by using the Kimura-2-parameter model with a discrete gamma distribution (K2 + G). Bootstrap support values were calculated using 1000 replicates. To estimate the average evolutionary distance, we used the Maximum Composite Likelihood method with 1000 bootstrap replications. Further recombination analysis of the CVA6 whole genomes was performed by aligning the genome sequences using the Clustal Omega 1.2.2 multiple alignment tool. Subsequent maximum likelihood tree construction based on the 3Dpol region was carried using the MEGA X v10.0.5 software (K2 + G model), and 1000 bootstrap replicates were set to estimate support. Simplot + + v1.3 software was used to analyze the genomic sequence similarity of the CVA6 recombinant forms, with a 200 bp window moving in 20 nt steps using the Kimura distance model [23].

## Results

### Laboratory diagnosis of EV infections

Between 01 January 2020 and 31 December 2024 an overall number of 2,324 clinical samples were received for EV testing at the National Reference Laboratory for Enteroviruses in Budapest, with the lowest number (360 and 366 tests) requested in 2020 and 2021, respectively, and the most (568 tests) in both 2022 and 2024 (data not shown). In 2023, 462 samples were processed for analysis. Of these, 173 (7.44% positivity rate) samples corresponding to 125 patients were found to be EV-positive. The median age of the positive patients was 7 years (in the range of 0–58 years).

**Fig. 1:**
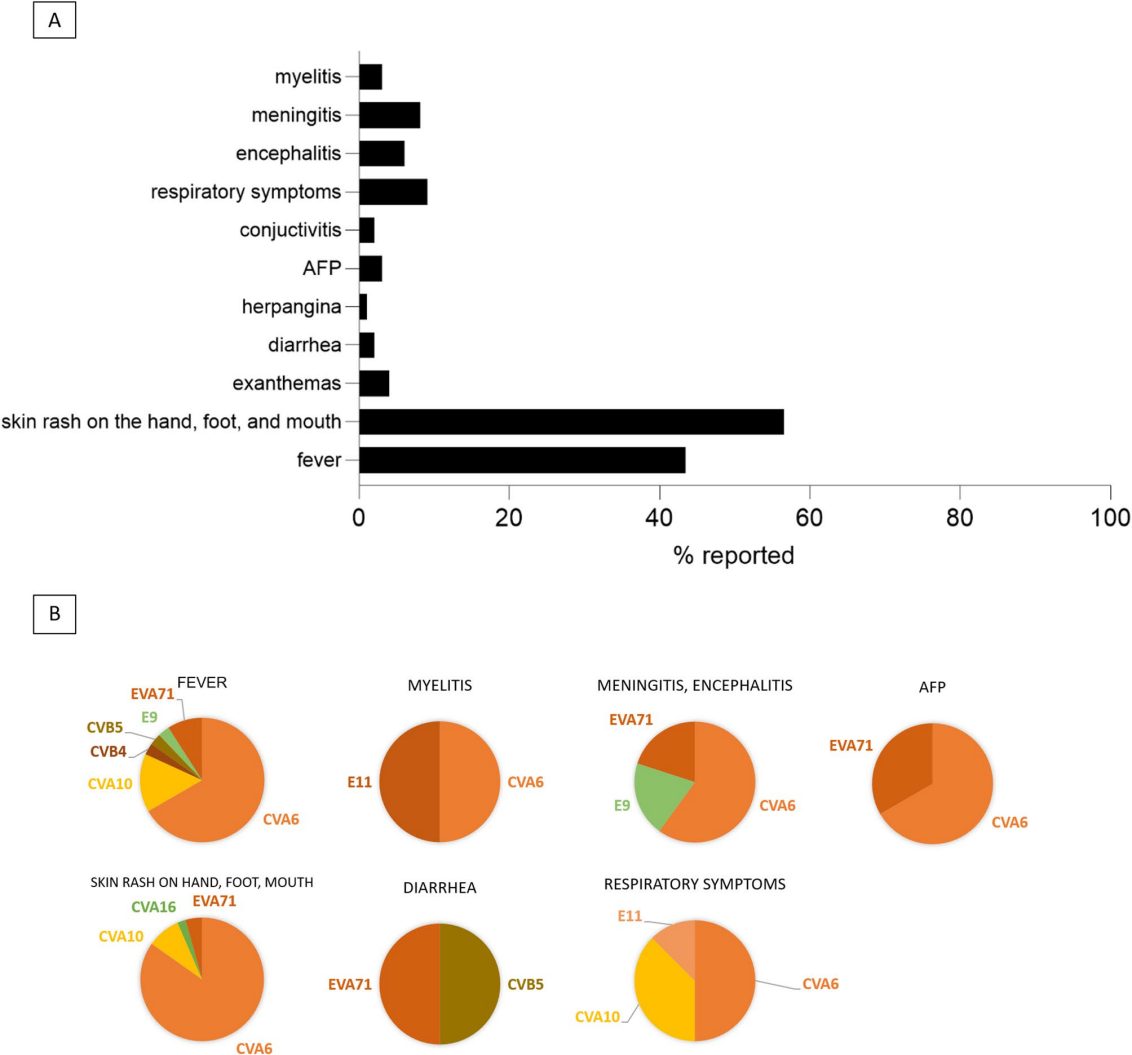
(**A**) Leading symptoms reported among EV-infected patients (*n* = 99). (**B**) Most reported symptoms by causative EV genotype

Symptoms were reported in the case of 99 (79.2%) EV-positive patients. Leading symptoms were fever, skin rash on the hand, foot, and mouth, herpangina, diarrhea, exanthemas, AFP, conjunctivitis, sore throat or other mild respiratory symptoms, encephalitis, meningitis, and myelitis (Fig. 1A). More severe symptoms, including meningitis, encephalitis, flaccid paralysis and myelitis were associated with EVA71, CVA6 and E9 infections. Respiratory symptoms were reported in the case of E11, CVA10 and CVA6 infections, while diarrhea was associated with EVA71 and CVB5 genotypes (Fig. 1B).

**Fig. 2:**
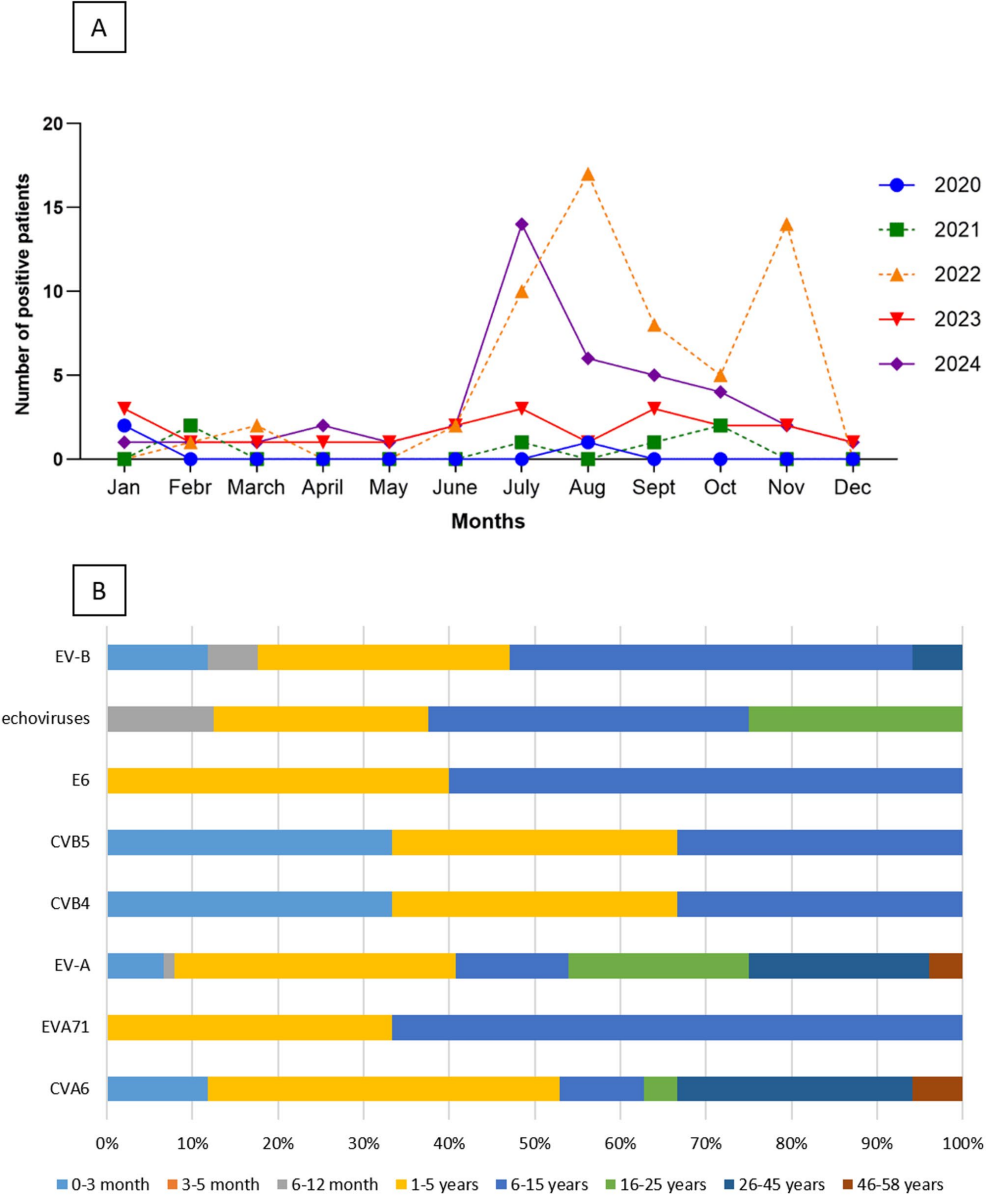
Distribution of diagnosed EV-positivity. (**A**) Temporal distribution (number of positive patients by month of sample collection, *n* = 125). Years of the study period (2020–2024) are indicated with different colors. (**B**) Age distribution of confirmed infections by identified genotypes and species

Temporal distribution of the diagnosed EV-positive cases shows strong seasonality with increased number of positivity during the summer and early-autumn periods (from July to November, Fig. 2A). During the first year following the COVID lockdown only a slight, non-significant increase was observed in the proportion of positive specimens. In 2020 (May 2020– April 2021), 2 out of 317 specimens tested positive (0.63%), followed by 6 of 416 (1.44%) in 2021 (May 2021– April 2022). The proportion of positive specimens increased notably two years after easing the lockdowns. After May 2022, a significantly increasing trend in EV detection was observed (61 positives out of 572 tests; 10.66%; *p* < 0.0001). In 2023 (May 2023 - April 2024), there was a decrease compared to the second year (20 positives out of 474; 4.22%), although EV detection levels remained significantly higher compared to the lockdown period (*p* = 0.0017). Accordingly, the lowest number of confirmed cases was in 2020 (*n* = 3) and 2021 (*n* = 4) during the COVID-19 lockdown, and the highest number of patients was identified in 2022 (*n* = 59) after easing the restrictions. In 2023 and 2024, an overall number of 22 and 39 cases were diagnosed, respectively.

The ages of patients with confirmed EV infections ranged from 0 to 58 years, with a median age of 7 years. The majority of cases occurred in children aged 1 to 5 years (29.6%, *n* = 37). Infants under 1 year represented 14.4% of all cases (*n* = 18) and were mostly attributed to CVA6, CVA10, E11, CVB4 and CVB5 genotypes. In the 1–5 years and 6–15 years age groups, genotypes of both EV-A and EV-B species were responsible for the infections. In contrast, among older age groups (26–45 years and 45–58 years), enterovirus A was more commonly associated with the infections, with only a single case of an EV-B species identified in the 26–45 years group (Fig. 2B). In accordance with the descriptive analysis, the SDI confirmed that the prevalence of the identified EV genotypes shows age-dependency (Supplementary Materials Table 3).

**Fig. 3:**
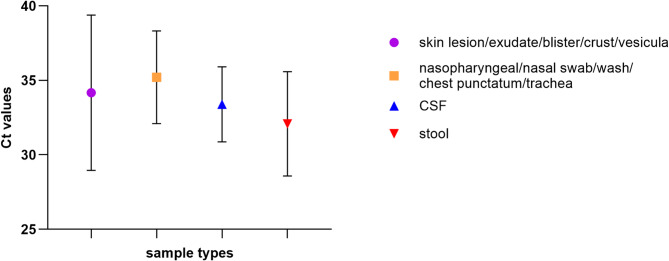
Ct vales of the EV-positive samples by specimen type detected during the study period (2020–2024, *n* = 125). EV positivity was reported under Ct 39. Error bars represent Ct range obtained during the study

The most frequent sample types received and tested positive were stool (41.76%, 71 out of 173 samples), nasopharyngeal swab/wash and other respiratory samples (34.12%, 58 out of total 173 positive), skin lesions/blisters and vesicular fluid (11.18%, 19 out of 170) and CSF (12.94%, 22 out of 173). The highest viral loads (corresponding to the lowest Ct values) were detected in stool samples (average Ct 32.08), while the lowest viral loads (corresponding to the highest Ct values) were seen in respiratory samples (average Ct 34.20; Fig. 3A). In the case of neurological involvement, CSF was shown to be an adequate specimen type for EV testing with the second highest average viral loads (average Ct 33.39, Fig. 3).

Virus isolation in RD cells was attempted from 75 clinical specimens with Ct values ≤ 37.0. Isolation was successful in the case of 24 samples, mostly from stool (17, 70.83%), vesicular fluid (3, 12.50%) and CSF (3, 12.50%). Interestingly, there was no significant difference between the initial Ct values of the RD-positive and negative samples (*P* = 0.4002). The median Ct value was 30.54 among clinical specimens that were successfully isolated, compared to the median Ct 31.15 among samples with unsuccessful isolation.

### Genotyping

SISPA-Seq and/or 5’UTR amplicon sequencing was performed directly from the clinical samples or cell culture isolates corresponding to 105 patients. Samples of 20 patients were excluded due to extremely low viral load (Ct value ≥ 38.0). In the case of 18 patients, the determination of underlying EV genotype was unsuccessful. The most frequent EV genotype identified during the study period was CVA6 (52.80%). Frequency of other types were significantly lower (CVA10 4.80%; CVB4 2.40%; CVB5 2.40%; CVA16 0.80%, E6 4.00%; E11 0.80%; E18 0.80%; E9 0.70%, and EVA71 2.40%). We were able to assemble 21 whole genome or capsid sequences and 33 additional VP1 sequences. The complete list of generated sequences and Genbank accessions numbers are presented in the Supplementary Materials Table 1. Partial 5’UTR sequencing was successful in 33 cases.

**Fig. 4:**
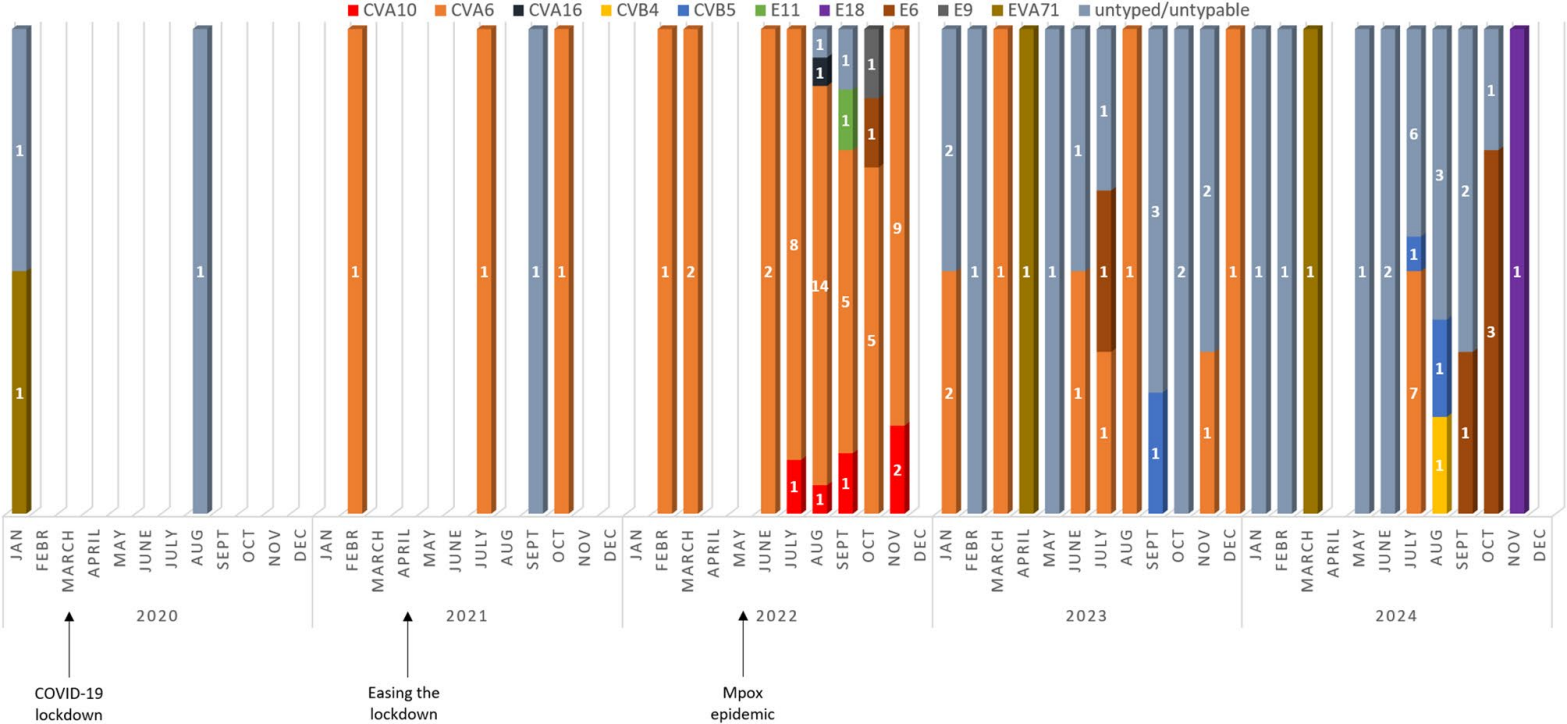
Temporal distribution of identified circulating EV types between 2020 and 2024 by the month of sample collection

Distribution of identified EV genotypes increased after easing the COVID-19 lockdown restrictions in Hungary (Fig. 4). Only a few cases were identified during the lockdown (*n* = 2, between March 2020 and April 2021). This phenomenon is attributed to the limited capacity of sample collection and testing during the pandemic and the reallocation of diagnostic resources, and the probable consequence of the reduction in personal contacts due social distancing. During 2021, we detected exclusively CVA6 in the clinical samples. During the summer season of 2022, a significant increase in the number of EV-positive cases were seen (Fig. 2) and additional EV genotypes emerged such as CVA10, CVA16, E9 and E11. A high increase in the emergence of EV-B species was identified in the seasonal periods of 2023 (18.18% of infections) and 2024 (46.67% infections), compared to previous years, when EV-A species accounted for the majority of the cases. A cluster of CVA10 cases emerged in the season of the 2022, between July and November (*n* = 5). Interestingly, we did not detect any further CVA10 circulation in the following years. Our findings are further supported by the SDI analysis; diversity decreased in the post-lockdown period, during which only the CVA6 genotype was detected (H’ 0.261) compared to the initial H’ value of 0.401 during the lockdown. However, it later increased relative to the lockdown period (H’ 0.502 in the 2022 season, H’ 0.487 in the 2023 season) (Supplementary Materials Table 2).

The highest number of EV-positive patients was identified in 2022 (*n* = 59), most of them with rash and skin lesions consistent with HFMD. The vast majority of the infections were attributed to CVA6. Due to the Mpox epidemic in 2022, the number of samples requested for testing obtained from patients with skin rash and lesions increased significantly countrywide (data not shown).

During the study period, we detected EV-positivity in 22 CSF samples corresponding to 20 clinical cases presented with neurological involvement (encephalitis, meningitis, fever, hypersomnia, and disturbance of consciousness). The average Ct value in the CSF specimens was 33.39 (Fig. 3). EV genotype was determined in 10 cases with CVA6, CVB5, CVB4, E9, E11, and E18 being the cause of infection. The median age of affected patients was 16 years (in the range of 0–36 years).

In the frame of the national poliovirus AFP surveillance, an overall number of 55 patients (2020: 8, 2021: 8, 2022: 14, 2023: 9 and 2024: 16) were tested between 2020 and 2024. Among them, three patients were non-polio EV positive. Two patients were CVA6 positive in 2022, and one infection was attributed to EVA71 infection, diagnosed in 2024. The leading symptoms were high fever, conjunctivitis, encephalitis, paralysis, nausea, headache, and migrating joint pain. The median age of affected patients was 2 years (ranging between 2 and 11 years).

### Phylogenetic analysis of the circulating CVA6, EVA71, CVB5, CVB4 and E6 strains

#### Coxsackievirus A6

**Fig. 5:**
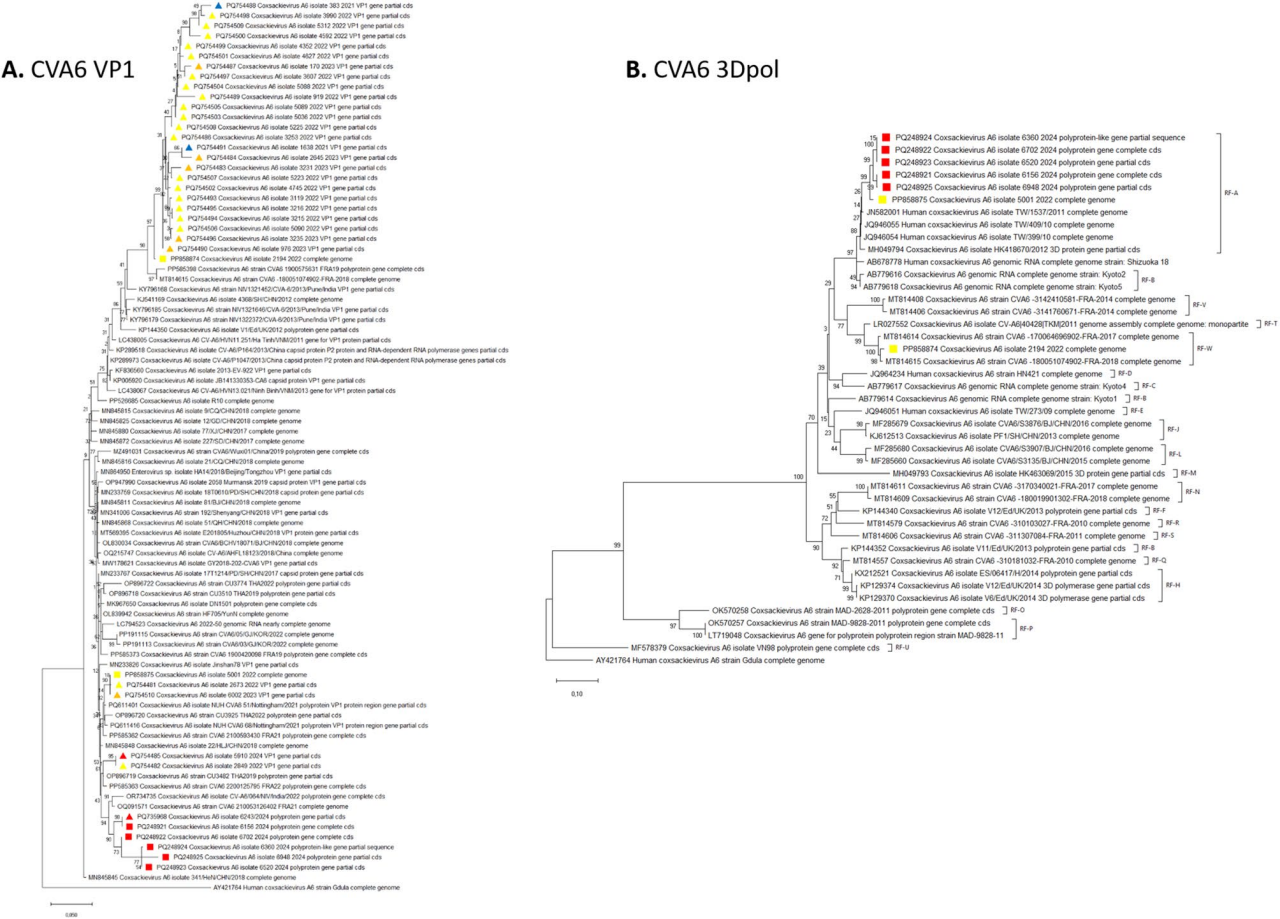
Maximum likelihood phylogenetic tree (K2 + G model) of the newly generated CVA6 strains based on the (**A**) VP1 region, and the (**B**) 3Dpol region. Isolates from 2021, 2022, 2023, and 2024 are denoted by blue, yellow, orange and red colors, respectively. Assembled whole genomes are indicated with squares

Phylogenetic analysis of the Hungarian CVA6 VP1 sequences (*n* = 37) revealed two distinct clusters (Fig. 5A). Strains circulating between 2020 and 2023 grouped together and showed a close relationship with French isolates from 2018 to 2019, which were circulating prior to the COVID-19 lockdowns. CVA6 VP1 sequences from 2024 formed a separate sub-cluster, displaying similarities with French and Indian strains from 2022 to 2023. All newly generated VP1sequences are classified under sub-cluster D3a.

Based on the phylogenetic tree constructed from the 3Dpol region (Fig. 5B), which includes our newly generated Hungarian sequences (*n* = 7) alongside previously described CVA6 recombinant forms (RFs) from around the world [24–26], we observed that all 2024 strains, along with one strain isolated in 2022, belong to RF-A, while PP858874 is classified under RF-W. This sequence also clusters separately from the 2024 strains in the VP1 region (Fig. 5A). Results of the similarity plotting is shown in Supplementary Materials Fig. 1.

#### Enterovirus A71

Phylogenetic analysis of the EVA71 VP1 gene sequences (Supplementary Materials Fig. 2) demonstrated that isolate PP858876 (2020) shared the highest similarity with a German EVA71 strain from 2019 (Genbank accession No. MN397897), which emerged prior to the COVID-19 pandemic, during a period with increased EVA71 incidence in Germany. The VP1 sequence of isolate PP858877, derived from the stool sample of an AFP case in 2024, clustered closely with a Russian EVA71 strain isolated from the fecal sample of a child diagnosed meningoencephalitis in 2022 (Genbank accession No. OP947994). The pairwise distance between PP858876 and PP858877 VP1 regions was calculated to be 0.041, while the overall average distance among the EVA71 VP1 sequences included in the phylogenetic analysis was 0.02 (SD = 0.0). Based on VP1 classification both identified EVA71 strains belong to C1-like sub-genogroup.

#### Coxsackie B5 and B4

During our study period, CVB5 was detected in both 2023 and 2024, while CVB4 was identified in 2024 following the COVID-19 pandemic. No cases attributed to these genotypes were observed in Hungary during the lockdown. Phylogenetic analysis of the VP1 gene (Supplementary Materials Fig. 3B) from the CVB4 isolates (PQ335016 and PQ248920) revealed a close genetic relationship with CVB4 strains isolated in France in 2016 (Genbank accession Nos. PP558469-70), 2020 (Genbank accession No. PP558477), and in the UK in 2018 (Genbank accession No. MT641411) (Supplementary Materials Fig. 3). The average pairwise distance between the study strains (PQ335016 and PQ248920) was 0.0147, while the overall distance among all CVB4 VP1 sequences in the analysis was 0.14 (SD = 0.01). The three CVB5 strains identified during the study period (PP858873, PQ735967, PQ211274) clustered together in the phylogenetic tree based on the VP1 gene analysis. The closest relationship was observed with CVB5 strains found in Thailand in 2023 and Italy in 2022. The overall genetic distance among all sequences in the phylogenetic analysis was 0.13 (SD = 0.01).

#### Echovirus E6

During and directly after easing the COVID-19 lockdown measures (between 2020 and 2022), no infections attributable to E6 was detected. Phylogenetic analysis of the VP1 region revealed that a strain isolated in 2023 (PP887984) clustered with Russian strains from AFP cases in 2018. The newly characterized Hungarian sequences of the 2024 strains clustered together and showed close similarity to a French strain from 2022 (Supplementary Materials Fig. 4). The overall genetic diversity among the sequences included in the phylogenetic tree analysis was 0.17 (SD = 0.01).

## Discussion

Due to the strict public health measures implemented globally to control the transmission of SARS-CoV-2 in 2020, the transmission of other viral respiratory infections significantly decreased. Efforts to limit person-to-person contact, alongside the decline in the use of public transport during the lockdowns, contributed to a decrease in the incidence of other infectious diseases, including enterovirus infections [5]. To investigate potential epidemiological differences attributed to the bottleneck effect caused by temporarily disrupted transmission chains, we analyzed the EV genotypes responsible for infections in Hungary from January 2020 to December 2024, covering the period during and after the COVID-19 lockdowns.

During the study period, a total of 2,324 clinical samples from symptomatic individuals were submitted for EV testing at the National Reference Laboratory for Enteroviruses in Budapest. Of these, 173 samples, corresponding to 125 individual cases, were found to be EV-positive. This relatively low proportion of positive cases reflects the reduced circulation of enteroviruses during the early phases of the COVID-19 pandemic, as public health measures likely led to a temporary decline in viral transmission.

Following the easing of global restrictions in 2021, multiple countries reported an increase in enterovirus infection cases [5]. Poland reported an increase in the circulation of E11 post-lockdown, with the emergence of a highly pathogenic variant responsible for severe neonatal infections. Between 2017 and 2023, E11 strains exhibited high genetic diversity, and by 2022–2023, a new variant associated with increased morbidity and mortality was detected, underscoring the potential for significant shifts in enterovirus pathogenicity following pandemic-related disruptions [27]. Additionally, a regional study conducted in the UK between 2018 and 2023 revealed an increase in genetic diversity among circulating CVA6 strains [28]. Similarly, in our study, the genetic diversity among EV genotypes increased significantly following the easing of the COVID-19 lockdowns. This increase in diversity is particularly notable when compared to the limited data available for the year 2020, when only 360 samples were received for testing. The restricted sample size during this period likely limited our ability to capture the full spectrum of circulating genotypes due to reduced statistical power and falsely increased variability in EV genotype and patients age distribution by sampling bias. However, with the rise in sample submissions to over 550 after 2021, a broader representation of circulating EV genotypes was obtained, highlighting the post-lockdown increase in genetic diversity. Although direct comparison of EV genotype distributions before and after the COVID-19 lockdown are not possible, as data on EV genotypes prior to the lockdowns were not directly available to us, the diversity of EV types appears to have increased after easing the lockdown. We observed a broader peak of EV seasonality in 2022, compared to following years, that may reflect, in part, an increased number of samples submitted during the concurrent mpox outbreak in 2022, potentially leading to higher EV differential detection. It is also possible that the co-circulation of multiple EV genotypes and shifts in human behavior and mobility, following COVID-19 restrictions played important role. A marked increase in the emergence of EV B species (CVB4, CVB5, and echoviruses) was observed in the seasonal periods of 2023 (18.18% of infections) and 2024 (46.67% of infections), compared to previous years when EV A species (CVA6, CVA10, CVA16, EVA71) were predominantly responsible for the majority of cases. Notably, a cluster of E6 cases emerged in the second half of 2024, marking a resurgence of this genotype. Prior to the COVID-19 pandemic, E6 had been one of the dominant circulating strains in Hungary, along with E30, E11, and E9 [29]. The re-emergence of E6 and other echoviruses underscores the dynamic nature of EV circulation, with certain genotypes regaining prominence as pandemic-related disruptions in transmission gradually decrease.

CVA6 has been recognized as a major cause of HFMD globally since 2008, with significant prevalence in both Europe and the United States over the past decade [28]. While enterovirus infections have traditionally been viewed as predominantly summer infections due to their seasonal nature, this oversimplification does not hold true for all enterovirus types [30]. In line with Joyce et al., we also observed that CVA6 circulates during both summer and autumn, with peak activity between June and November (Fig. 2). Since 2014, CVA6 has accounted for approximately 50% of enterovirus infections in Hungary [29], but this proportion appears to have decreased following the pandemic (Fig. 3). Based on full-genome sequence-based recombination analysis (Supplementary Materials Fig. 1) and phylogenetic trees (Fig. 5), including the complete genome sequences of additional internationally identified CVA6 strains, the emergence of a novel recombinant form during the spread of CVA6 in Hungary is unlikely. Since 2024, infections have been predominantly caused by the RF-A form, consistent with international trends [26, 31]. A study conducted in the Netherlands observed a decrease in genetic diversity among five commonly detected EV genotypes — EV-D68, E11, CVA6, CVB5, and CVA2 — after the easing of lockdown measures. This reduction was characterized by a higher sequence homology among circulating strains and an increase in the median age of patients, particularly for E11 and CVB5 infections. The authors suggest that the diminished transmission during lockdowns may have led to a bottleneck effect, reducing viral diversity [5]. Similarly, research from Thailand reported a significant decline in enterovirus prevalence and genotype diversity during the COVID-19 pandemic among patients with acute gastroenteritis. The study attributes this decrease to enhanced hygiene practices, social distancing, and mask-wearing, which likely curtailed enteric virus transmissions as well [32].

Most dominant symptoms reported during the study period were fever, skin rash on the hand, foot, and mouth, herpangina, diarrhea, exanthemas, AFP, conjunctivitis, sore throat or other mild respiratory symptoms, encephalitis, meningitis, and myelitis (Fig. 1). The median age of patients observed in our study aligns with previous findings worldwide [29, 33, 34]. Between 2010 and 2018, the median age of confirmed EV patients was reported as 5 years in Hungary, with the highest incidence occurring in the 1–5 and 6–15 age groups [29]. In contrast, during the period of 2020 to 2024, the median age of affected individuals increased to 7 years. Despite this shift, the most affected age groups remained consistent, with the younger populations (1–5 and 6–15 years old) continuing to show the highest incidence of infection. This slight increase in median age may be partly attributable to the enhanced number of samples received from older individuals during the 2022 mpox outbreak, when both mpox and enterovirus infections were screened for differential diagnosis purposes.

Virus isolation was performed in accordance with WHO guidelines for 75 positive clinical samples using RD cells [35]. This procedure was successful for 24 samples (32.0%), with stool samples being the most reliable source, accounting for 70.83% of successful isolations. These findings align with previous studies [14, 29, 30, 36], reinforcing the importance of stool as a key sample type for enterovirus diagnosis. Additionally, the highest viral loads, reflected by the lowest Ct values, were detected in stool samples (average Ct = 32.08). This further highlights the diagnostic value of stool specimens, as they are not only more likely to yield successful virus isolation but also exhibit higher viral replication levels, making them critical for accurate detection and subsequent molecular analysis.

## Conclusions

In conclusion, our study highlights the evolving dynamics of enterovirus circulation in Hungary during and after the COVID-19 pandemic. The easing of lockdown measures was associated with increased genetic diversity among circulating enterovirus strains, likely reflecting shifts in transmission patterns. The increase in the median age of affected individuals and the continued dominance of younger age groups further illustrate the changing landscape of enterovirus infections. These observations emphasize the importance of continued surveillance and sequencing efforts, which are essential for tracking viral evolution, identifying emerging strains, and understanding how public health interventions influence viral transmission.

## Electronic supplementary material

Below is the link to the electronic supplementary material.

Supplementary Material 1

## Data Availability

Sequence data that support the findings of this study have been published in the National Center for Biotechnology Information (NCBI) repository. Corresponding accession numbers are included in the Supplementary materials.
