# Supplementary material for: Evaluating enterovirus diversity among symptomatic patients in Hungary during and after easing the COVID-19 lockdown
Source: Virol J. 2025 Jun 24;22:204. doi: 10.1186/s12985-025-02835-2 (PMC12186400; doi:10.1186/s12985-025-02835-2)
Supplement: Supplementary file 1 — Supplementary Material 1 [file 12985_2025_2835_MOESM1_ESM.docx]

Supplementary materials

Title: **Evaluating Enterovirus Diversity among Symptomatic Patients in Hungary During and After Easing the COVID-19 Lockdown**

Nóra Deézsi-Magyar^1,2*^, Gyula Zsidei^1^, Norbert Kiss^1^, Bereniké Novák^1^, Marianna Mezősi-Csaplár^1^, Katalin Réka Tarcsai^1^, Adrienne Lukács^1^, Erzsébet Barcsay^1^, Katalin Szomor^1+^ and Mária Takács^1,3+*^

1 Department of Microbiological Reference Laboratories, National Center for Public Health and Pharmacy, 1097 Budapest, Hungary

2 School of PhD Studies, Semmelweis University, 1089 Budapest, Hungary

3 Institute of Medical Microbiology, Semmelweis University, 1089 Budapest, Hungary.

*Corresponding authors

+These authors contributed equally to this work.

Correspondence: magyar.nora@nngyk.gov.hu, takacs.maria@semmelweis.hu

**Supplementary Table 1.**

| **Year of collection** | **Sample ID** | **Ct value** | **Sequence region** | **EV genotype** | **Genbank Accession No.** |
| --- | --- | --- | --- | --- | --- |
| 2020 | 106/2020 | 32,23 | WGS | EVA71 | PP858876 |
| 2021 | 383/2021 | 32,72 | VP1 | CVA6 | PQ754488 |
| 2021 | 1638/2021 | 33,86 | VP1 | CVA6 | PQ754491 |
| 2022 | 919/2022 | 33,49 | VP1 | CVA6 | PQ754489 |
| 2022 | 2194/2022 | 29,06 | WGS | CVA6 | PP858874 |
| 2022 | 2673/2022 | 32,55 | partial VP1 | CVA6 | PQ754481 |
| 2022 | 2849/2022 | 28,47 | partial VP1 | CVA6 | PQ754482 |
| 2022 | 3104/2022 | 35,56 | VP1 | CVA6 | PQ754492 |
| 2022 | 3119/2022 | 24,71 | VP1 | CVA6 | PQ754493 |
| 2022 | 3215/2022 | 33,48 | VP1 | CVA6 | PQ754494 |
| 2022 | 3216/2022 | 30,97 | VP1 | CVA6 | PQ754495 |
| 2022 | 3253/2022 | 31,64 | partial VP1 | CVA6 | PQ754486 |
| 2022 | 3607/2022 | 33,2 | VP1 | CVA6 | PQ754497 |
| 2022 | 3712/2022 | >38,0 | WGS | E-11 | PQ248926 |
| 2022 | 3953/2022 | >38,0 | WGS | CVA10 | PP858872 |
| 2022 | 3990/2022 | 33,42 | VP1 | CVA6 | PQ754498 |
| 2022 | 4352/2022 | 29,5 | VP1 | CVA6 | PQ754499 |
| 2022 | 4592/2022 | 34,46 | VP1 | CVA6 | PQ754500 |
| 2022 | 4627/2022 | 26,98 | VP1 | CVA6 | PQ754501 |
| 2022 | 4745/2022 | 30,14 | VP1 | CVA6 | PQ754502 |
| 2022 | 5001/2022 | 29,6 | WGS | CVA6 | PP858875 |
| 2022 | 5036/2022 | 33,61 | VP1 | CVA6 | PQ754503 |
| 2022 | 5088/2022 | 33,8 | VP1 | CVA6 | PQ754504 |
| 2022 | 5089/2022 | 33,75 | VP1 | CVA6 | PQ754505 |
| 2022 | 5090/2022 | 30,03 | VP1 | CVA6 | PQ754506 |
| 2022 | 5223/2022 | 30,44 | VP1 | CVA6 | PQ754507 |
| 2022 | 5225/2022 | 32,03 | VP1 | CVA6 | PQ754508 |
| 2022 | 5312/2022 | 32,58 | VP1 | CVA6 | PQ754509 |
| 2023 | 170/2023 | 30,78 | VP1 | CVA6 | PQ754487 |
| 2023 | 976/2023 | 27,09 | VP1 | CVA6 | PQ754490 |
| 2023 | 2645/2023 | 32,24 | partial VP1 | CVA6 | PQ754484 |
| 2023 | 3231/2023 | 31,29 | partial VP1 | CVA6 | PQ754483 |
| 2023 | 3235/2023 | 32,9 | partial VP1 | CVA6 | PQ754496 |
| 2023 | 3535/2023 | 28,56 | WGS | E-6 | PP887984 |
| 2023 | 5246/2023 | 31,66 | WGS | CVB5 | PP858873 |
| 2023 | 6002/2023 | 29,06 | VP1 | CVA6 | PQ754510 |
| 2024 | 2442/2023 | 28 | WGS | EVA71 | PP858877 |
| 2024 | 5910/2024 | 30,78 | partial VP1 | CVA6 | PQ754485 |
| 2024 | 6156/2024 | 24,08 | WGS | CVA6 | PQ248921 |
| 2024 | 6243/2024 | 31,95 | whole capsid | CVA6 | PQ735968 |
| 2024 | 6360/2024 | 35,04 | WGS | CVA6 | PQ248924 |
| 2024 | 6520/2024 | 33,15 | WGS | CVA6 | PQ248923 |
| 2024 | 6702/2024 | 27,25 | WGS | CVA6 | PQ248922 |
| 2024 | 6740/2024 | 30,98 | WGS | CVB4 | PQ335016 |
| 2024 | 6784/2024 | 31,68 | WGS | CVB5 | PQ211274 |
| 2024 | 6948/2024 | 25,75 | WGS | CVA6 | PQ248925 |
| 2024 | 6978/2024 | 30,07 | WGS | CVB5 | PQ735967 |
| 2024 | 7169/2024 | 32,03 | WGS | CVB4 | PQ248920 |
| 2024 | 8049/2024 | 28,24 | partial VP1 | E-6 | PQ759017 |
| 2024 | 10710/2024 | 33,09 | partial VP1 | E-18 | PQ759018 |
| 2024 | 9550/2024 | 33,17 | WGS | E-6 | PQ785998 |
| 2024 | 10106/2024 | 27,44 | WGS | E-6 | PQ785999 |
| 2024 | 10272/2024 | 24,72 | WGS | E-6 | PQ785997 |

**
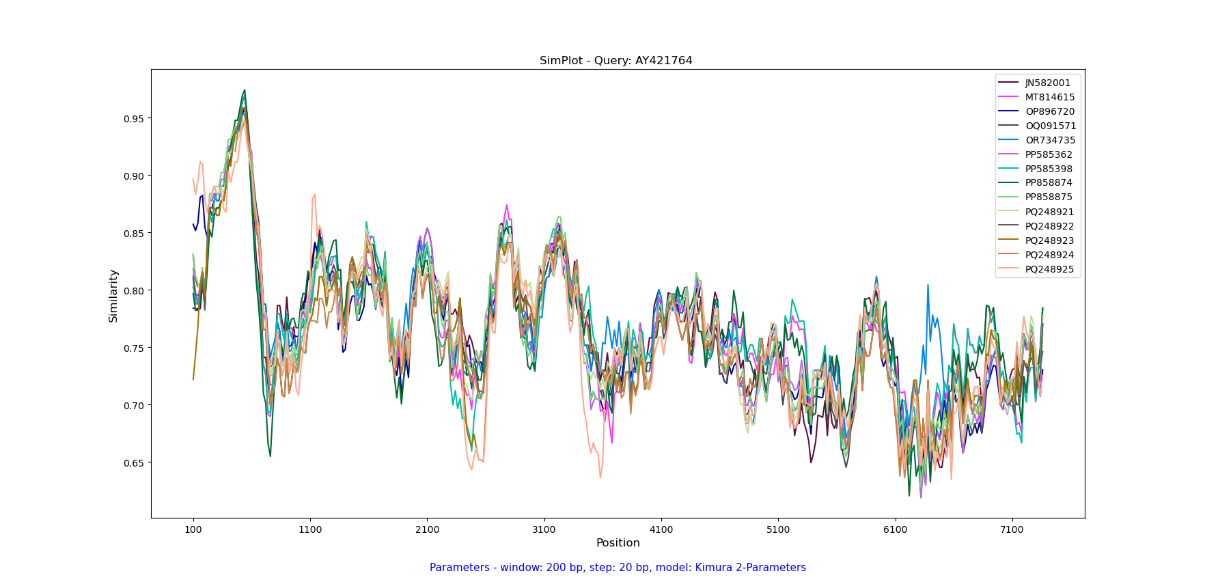
**

**Supplementary Figure 1.** Plot of similarity of the study CVA6 strains and closely related strains. Reference sequence CVA6 Gdula (AY421764) was set as query. Analysis was conducted by using SimPlot++ v1.3 (Kimura distance model, window size 200 bp moving in 20 nt steps).

**Supplementary Table 2. Shannon Diversity index on the effect of seasonality and easing the COVID-lockdown in Hungary on EV diversity**

|  | ASDI (H') |
| --- | --- |
| 2020 May - 2021 April (during lockdown) | 0.401 |
| Season of 2021 (post lockdown) | 0.261 |
| Season of 2022 | 0.502 |
| Season of 2023 | 0.487 |

**Supplementary Table 3. Shannon Diversity index on the effect of patient age on EV diversity**

|  | ASDI (H') |
| --- | --- |
| 0-3 months | 0.694 |
| 4-5 months | n.a |
| 6-12 months | 0.678 |
| 1-5 years | 0.669 |
| 6-15 years | 0.858 |
| 16-25 years | 0.305 |
| 26-45 years | 0.380 |
| 46-58 years | 0.306 |


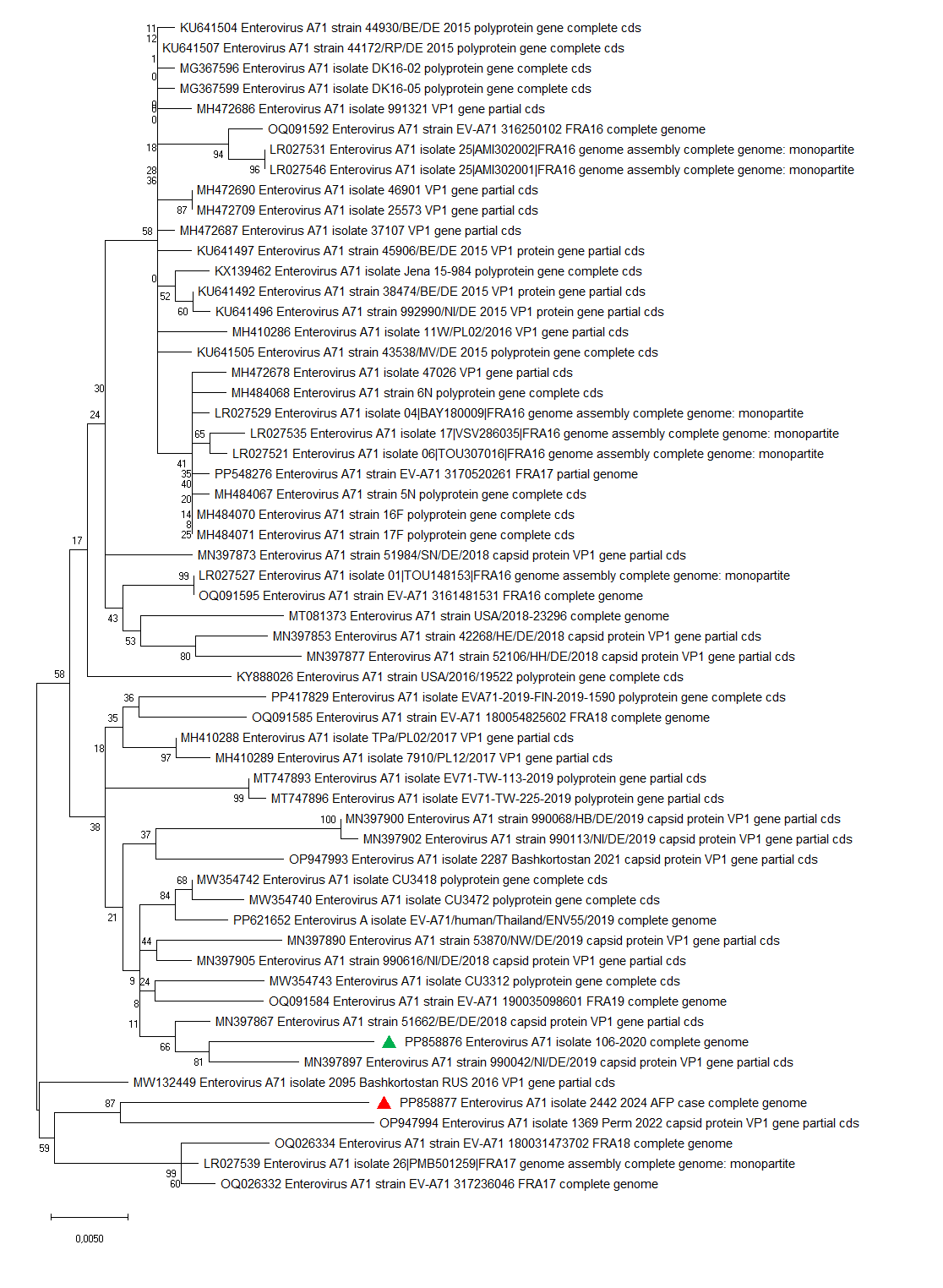


**Supplementary Figure 2. Maximum likelihood phylogenetic tree (K2+G model) of EVA71 VP1 sequences, illustrating the phylogenetic relationships between the EVA71 isolates detected during our study period in Hungary and other strains reported worldwide.** PP858876 isolated in 2020 (before COVID-19 pandemic lockdowns) and PP858877 isolated in 2024 (after easing the lockdowns) are highlighted with green and red triangles, respectively. RRID:SCR_000667

**
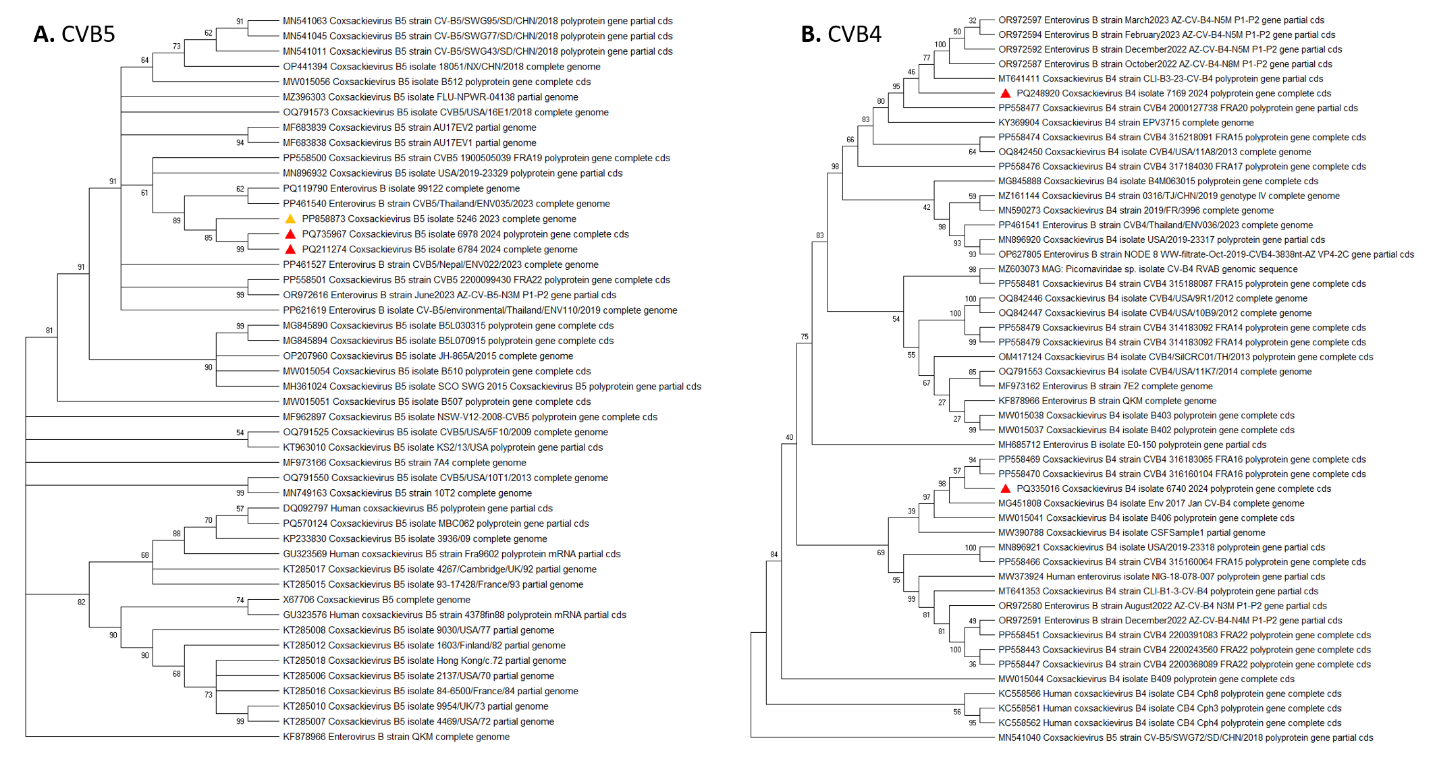
**

**Supplementary Figure 3.** **Maximum likelihood bootstrap consensus phylogenetic tree (K2+G model) of the Hungarian CVB5 (A) and CVB4 (B) VP1 sequences alongside previously reported strains.** Isolates from 2023 are marked in orange, while strains from 2024 are highlighted with red triangles. RRID:SCR_000667


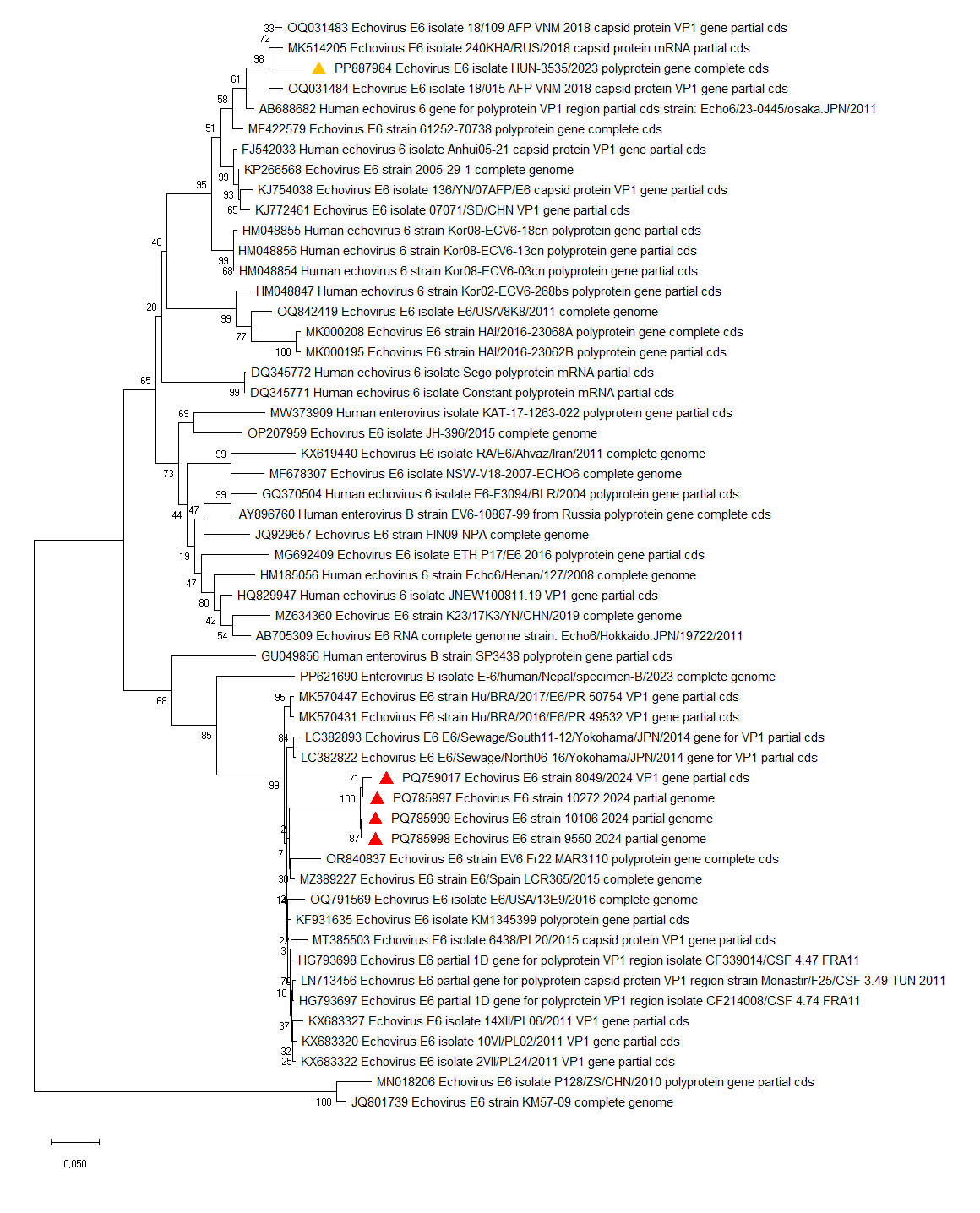


**Supplementary Figure 4. Maximum likelihood bootstrap consensus phylogenetic tree (K2+G model) of the Hungarian echovirus E-6 VP1 sequences alongside previously reported strains.** Isolates from 2023 are marked in orange, while strains from 2024 are highlighted with red triangles. RRID:SCR_000667
